# Alpha‐lipoic acid inhibits lung cancer growth via mTOR‐mediated autophagy inhibition

**DOI:** 10.1002/2211-5463.12820

**Published:** 2020-03-18

**Authors:** Peipei Peng, Xiaojin Zhang, Tao Qi, Hao Cheng, Qiuyue Kong, Li Liu, Xiaofei Cao, Zhengnian Ding

**Affiliations:** ^1^ Department of Anesthesiology First Affiliated Hospital with Nanjing Medical University China; ^2^ Department of Geriatrics Jiangsu Provincial Key Laboratory of Geriatrics First Affiliated Hospital with Nanjing Medical University China; ^3^ Laboratory of Targeted Intervention of Cardiovascular Disease Collaborative Innovation Center for Cardiovascular Disease Translational Medicine Nanjing Medical University China

**Keywords:** alpha‐lipoic acid, autophagy, lung cancer, mammalian target of rapamycin, rapamycin

## Abstract

Lung cancer is the leading cause of cancer‐related death, and there remains a need for novel therapies for this malignancy. Here, we examined the effects of alpha‐lipoic acid (LA), a drug used for treating human diabetic complications, on lung cancer growth. We report that LA limited lung cancer growth in xenograft mice and reduced lung cancer A549 cell viability. We observed autophagy activation in human lung cancers, and report that LA inactivated autophagy in A549 cells. In addition, LA activated mammalian target of rapamycin (mTOR)/p70S6K signaling. Inhibition of mTOR with rapamycin reversed LA‐induced inactivation of autophagy and abolished LA‐induced suppression of A549 cell viability. Altogether, the data suggest that LA exerts an anti‐lung cancer effect through mTOR‐mediated inhibition of autophagy, and thus LA may have therapeutic potential for lung cancer management.

## Graphical abstract

We report that alpha‐lipoic acid inhibits lung cancer growth *in vivo *and reduces lung cancer A549 cell viability *in vitro*. This anti‐lung cancer effect of alpha‐lipoic acid occurs via mammalian target of rapamycin‐mediated autophagy inhibition.

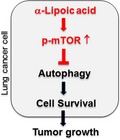

**Abbreviations**
CQ: chloroquine
LA: alpha‐lipoic acid
LC3: microtubule‐associated protein 1A/1B‐light chain 3
LDH: lactate dehydrogenase
mTOR: mammalian target of rapamycin
pEGFP–LC3: plasmid‐expressed rat LC3 fused to enhanced green fluorescent protein
Rap: rapamycin
SD: standard deviation

Lung cancer is the leading cause of cancer‐related death in humans worldwide, with an overall 5‐year survival rate of approximately 15% [1, 2, 3]. Despite advances in the diagnosis and treatment of lung cancer, patient survival has improved only modestly in the past 30 years [1, 2]. The dismal outcome mandates more investigation to identify a novel effective therapeutic approach for lung cancer management.

Recent evidence demonstrates that the cancer progression is closely related to autophagy activation [4, 5]. Autophagy is an evolutionarily conserved process for capturing and degrading dysfunctional and redundant cellular components to maintain the structural and metabolic homeostasis [6, 7, 8]. When cells encounter nutrient and energy deprivation and undergo apoptosis, autophagy is activated to promote cell survival [7]. Generally, cancer cells proliferate with abnormally high metabolic rate and thus require more involvement of autophagy for adaptive survival than normal cells [7, 9]. In supporting this, activation of autophagy has been shown to mediate the peritumoral monocyte‐induced cancer progression of human hepatocellular carcinoma, as well as the astrocyte‐induced promotion of breast cancer metastasis to the brain [5, 10]. Of particular interest, inhibition of autophagy has been shown to mediate the natural killer cell‐induced killing of non‐small‐cell lung cancer cells [4]. Therefore, targeting autophagy is suggested as a promising strategy for cancer therapy [4, 5].

Autophagy is a tightly and complicatedly regulated cellular event [11]. Among the multiple regulators, mammalian target of rapamycin (mTOR) plays a central role in initiation of autophagy [11]. mTOR forms complex 1 by binding with multiple companion proteins, such as mLST8, DEPTOR and Tti1/Tel2. In mammalian cells, mTOR forms complex 1 phosphorylates Ser758 (Ser757 in mouse) of unc‐51‐like kinase 1 (ULK1), preventing the interaction and phosphorylation of ULK1 by AMP‐activated protein kinase, which is essential for ULK1 activation for autophagy initiation and autophagosome formation [11, 12]. Thus, mTOR serves as a negative regulator for the initiation of autophagy.

Alpha‐lipoic acid (LA), a compound found in the human diet, has been used to treat variant human disorders. As examples, oral treatment with LA improves neuropathic symptoms and deficits, reduces triglycerides and improves quality of life in patients with painful diabetic neuropathy. Moreover, LA administration slows cognitive and functional decline in patients with Alzheimer’s disease and improves renal function in patients with diabetic nephropathy [13, 14]. Moreover, oral administration with LA shows no obvious adverse effect and even has been shown to be safe in pregnant women [15, 16, 17, 18]. We have previously demonstrated that LA attenuated cell injury induced by local anesthetics and improved cardiac dysfunction induced by endotoxin [19, 20]. However, little is known about whether LA could impact the growth of lung cancer in intact individuals.

To address this question, we established lung cancer models by implanting human lung cancer A549 cells in nude mice. We observed that oral LA administration markedly limited lung tumor growth in mice. The viability of lung cancer A549 cells was also suppressed by LA treatment. Molecular analysis revealed that the anti‐lung cancer effects of LA were mediated through promoting the mTOR‐mediated inhibition of autophagy. Our data suggest that besides the treatment for diabetic complications, LA might represent a viable therapeutic potential for the management of lung cancer in patients.

## Materials and methods

### Reagents

LA, chloroquine (CQ) and primary antibody for α‐Tubulin were obtained from Sigma‐Aldrich (St. Louis, MO, USA). Rapamycin (Rap) was from LC Laboratories (Woburn, MA, USA). Primary antibodies for Bcl‐2, Bax, phosphor‐Akt (p‐Akt^Ser473^), phosphor‐mTOR (p‐mTOR^Ser2448^) and total mTOR, phospho‐p70S6K (p‐p70S6K^Thr421/Ser424^) and p70S6K, VPS34, Beclin‐1, LC3‐II and p62 were obtained from Cell Signaling Technology (Beverly, MA, USA). Primary antibodies for ATG13 were obtained from San Ying Biotechnology (Wuhan, China). A primary antibody for glyceraldehyde‐3 phosphate dehydrogenase was from Bioworld Technology (Louis Park, MN, USA). Complete protease inhibitor cocktail was purchased from Roche (Mannheim, Germany). MTT [3‐(4,5‐dimethylthiazol‐2‐yl)‐2,5‐diphenyl‐tetrazolium bromide] reagent was from BioBasic, Inc. (Markham, ON, Canada). Cell‐Light™ EdU Apollo 567 In Vitro Kit was from RiboBio Technology (Guangzhou, China). SuperSignal West Pico chemiluminescent substrate was from Pierce (Rockford, IL, USA). FBS was from Life Technology (Grand Island, NY, USA). The plasmid‐expressed rat LC3 fused to enhanced green fluorescent protein (pEGFP–LC3) was provided by Addgene (Cambridge, MA, USA) [21].

### Human lung adenocarcinoma specimen collection

Human lung adenocarcinoma tissues and the adjacent noncancer tissues (>5 cm away from tumor lesion) were from patients undergoing pulmonary adenocarcinoma excision surgery at the Department of Thoracic Surgery, The First Affiliated Hospital of Nanjing Medical University. The patients without preoperative radiotherapy and chemotherapy were enrolled in this study. The adjacent normal lung tissues without inflammatory cell infiltration were verified by pathological examination using hematoxylin and eosin staining on paraffin‐embedded sections. Written informed consent was obtained from each subject. The Ethical Board of First Affiliated Hospital of Nanjing Medical University approved these studies (#2019‐SR‐279). All of the human study procedures were followed in accordance with the ethical standards of the responsible committee on human experimentation (institutional or regional) and with the Helsinki Declaration of 1975, as revised in 2000.

### Lung cancer xenograft in nude mice

Nude mice aged 6 weeks were implanted with A549 lung cancer cells (1 × 10^5^) through tail‐vein injection according to our previous study [3]. After cell implantation, mice were orally administrated with LA (50 mg·kg^−1^·day^−1^) in drinking water [22]. The cell‐implanted mice received normal drinking water served as vehicle controls. Eighteen days later, mice were sacrificed by overdose anaesthesia (pentobarbital sodium 150 mg·kg^−1^ intraperitoneal injection) and underwent cervical dislocation followed by examination of tumor numbers and tumor burdens. All experiments strictly conformed to the *Guide for the Care and Use of Laboratory Animals* published by the US National Institutes of Health (NIH Publication, 8th edition, 2011) and the international guidelines on the ethical use of animals. The animal care and experimental protocols were reviewed and approved by Nanjing University's Committee on Animal Care (#XG55).

### Cell culture and treatment

Human A549 lung cancer cells were cultured in a complete medium containing a Gibco™ (Grand Island, NY, USA) Dulbecco’s modified Eagle’s medium with 10% FBS. LA was administrated to the cells at the indicated dosages for the indicated durations. In mTOR inhibition experiments, cells were treated with Rap (500 nm) 1 h before LA (0.5 mm) treatment. In the CQ experiment, cells were treated with CQ (10 or 20 μm) 1 h before LA (0.5 mm) treatment.

### Examination GFP‐LC3 punctation

A549 cells, those grown on coverslips, were transiently transfected with the pEGFP–LC3 plasmid using the X‐tremeGENE HP DNA Transfection Reagent according to the manufacturer’s instructions. Twenty‐four hours later, the cells were stimulated with LA (0.5 mm) for 24 h and then fixed with 4% formaldehyde for 20 min. EGFP–LC3 puncta were observed and quantified using cellsens dimension 1.15 software (Olympus, Tokyo, Japan). The number of LC3 puncta per cell was counted in more than 20 random areas based on one batch of four independent experiments [23].

### Examination of cell viability

#### Morphology

Cells grown in 24‐well plates were treated with LA at the indicated concentrations for 72 h. Cellular morphology was examined using a phase‐contrast light microscope at magnifications of ×100 and ×400 (Zeiss Ltd., Oberkochen, Germany).

#### MTT assay

Cells grown in 96‐well plates were treated with LA at the indicated concentrations for 72 h. The viability of cells was examined using MTT assay according to our previous methods [24].

#### Lactate dehydrogenase measurement

After treatment with LA at the indicated concentrations for 72 h, medium of A549 cultures was collected for lactate dehydrogenase (LDH) activity measurement according to the manufacturer’s instructions.

#### 5‐Ethynyl‐2’‐deoxyuridine (EdU) assay

Cells grown in 96‐well plates were treated with LA at the indicated concentrations for 72 h and then were incubated with EdU for 2 h. Cell proliferation was indicated by EdU incorporation that was visualized by the assay kit according to the manufacturer’s instructions. Hoechst 33342 was used to counterstain the nuclei.

### Immunoblotting

A549 cells, those grown in 60‐mm dishes, were treated with LA at the indicated concentrations for 24 h. Cells treated with an equal volume of normal saline served as vehicle controls. After treatment, cells were harvested for immunoblotting analysis according to our previous studies [24]. The same membranes were also probed for glyceraldehyde‐3 phosphate dehydrogenase or α‐Tubulin for loading controls.

### Statistical analysis

Data are presented as means ± standard deviation (SD). Comparisons between groups were performed by Student’s two‐tailed unpaired *t‐*test or one‐way or two‐way ANOVA followed by Tukey’s *post hoc* test. Statistical significance was set at *P* < 0.05.

## Results

### LA limits lung cancer growth in mice

**Fig. 1:**
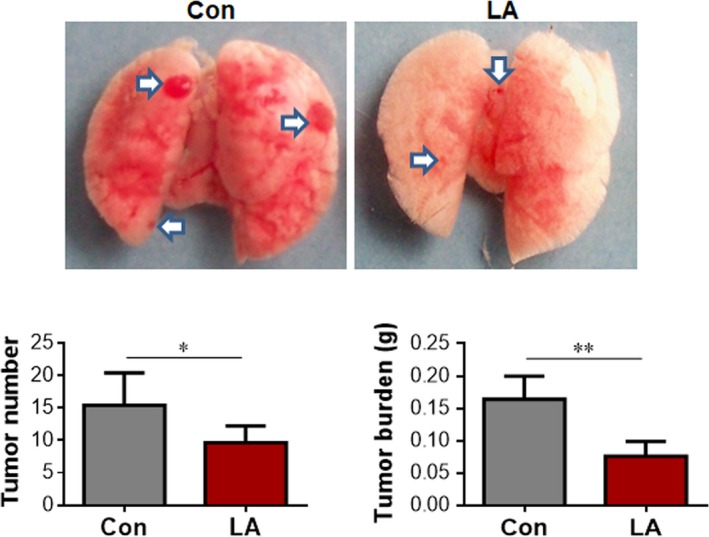
LA suppressed lung cancer growth in mice. Nude mice were implanted with A549 lung cancer cells intravenously and received LA administration. The implanted mice that received an equal volume of normal saline served as vehicle controls (Con). Eighteen days later, mice were sacrificed for examining the tumor nodule numbers and tumor weights. Arrows indicate the tumor nodules. **P < 0.01, *P < 0.05 by Student’s *t*‐test; error bars represent SD; *n* = 5 per group.

To determine the role of LA on lung cancer growth *in vivo*, we administrated LA orally to lung cancer nude mice that were established by implantation with lung adenocarcinoma A549 cells. The vehicle‐treated lung cancer mice served as controls. As shown in Fig. 1, LA treatment reduced tumor nodule numbers and tumor weights in lungs significantly by 37.7% and 60.6%, respectively, in comparison with vehicle‐treated control mice (*P < *0.01 or 0.05). The data suggest that LA exerted an anticancer effect on lung cancer growth in intact mice.

### LA decreases viability of A549 lung cancer cells

**Fig. 2:**
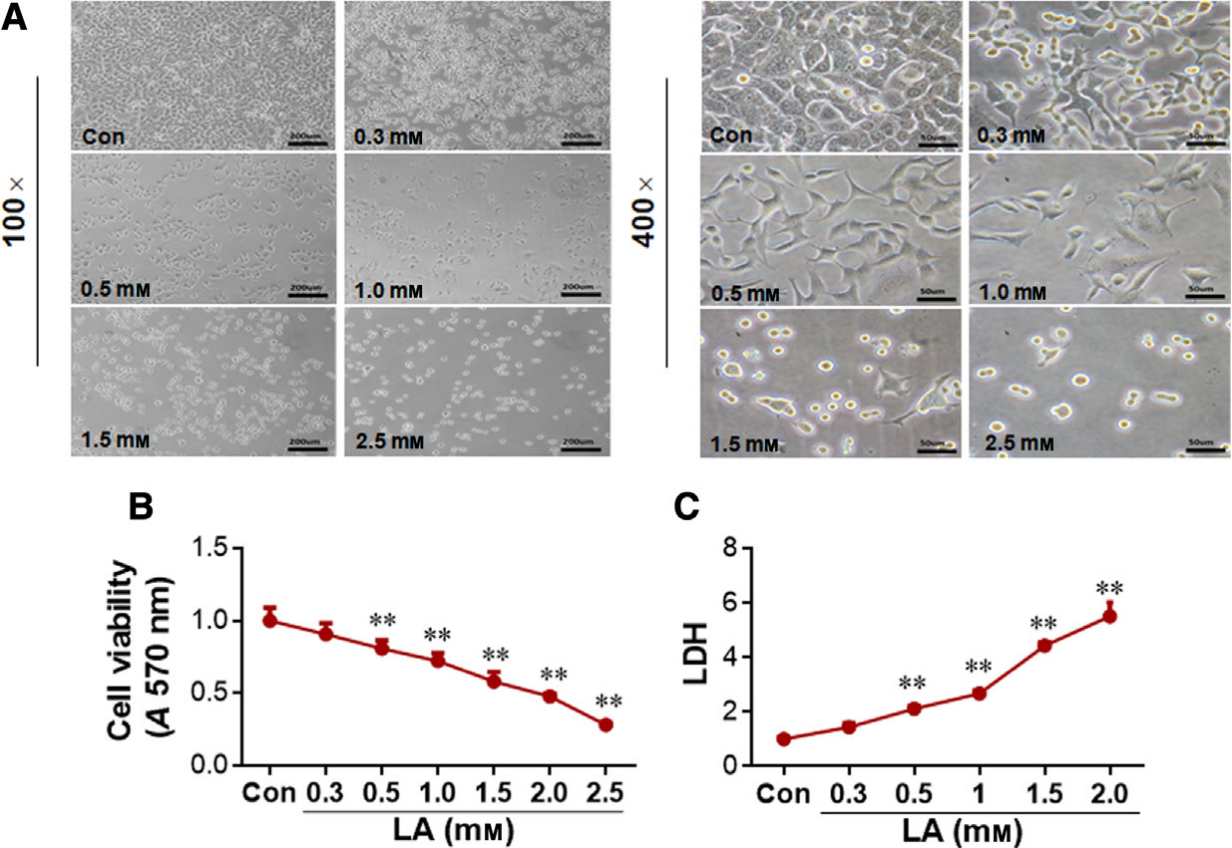
LA reduced viability of A549 cells. (A) Cell morphology. A549 cells were treated with LA at the indicated concentrations for 72 h. Normal saline‐treated cells served as vehicle controls (Con). Cell morphological alterations were observed using phase‐contrast microscope at original magnifications ×100 (left panels) and ×400 (right panels). Scale bars represents 200 µm (left panels); 50 µm (right panels). *n* = 4 per group. (B) MTT assay. A549 cells were treated with LA at the indicated concentrations for 72 h. Cell viability was evaluated by MTT assay. **P < 0.01 by one‐way ANOVA (versus vehicle controls); error bars represent SD; *n* = 4 per group. (C) LDH leakage. A549 cells were treated with LA at the indicated concentrations for 72 h. The LDH level was evaluated by LDH assay. **P < 0.01 by one‐way ANOVA (versus vehicle controls); error bars represent SD; *n* = 4 per group.

To investigate whether the inhibitory effect of LA on lung cancer growth is attributable to the reduction of cell viability, we treated A549 cells with LA at the indicated concentrations for 72 h. Morphological examination showed an obviously decreased cell density after treatment with 0.5, 1, 1.5 and 2.5 mm LA, respectively, when compared with the vehicle‐treated control cells (Fig. 2A). Moreover, the cells treated with LA with the doses of 0.5, 1, 1.5 and 2.5 mm exhibited shrunken shape and lost their cellular integrity compared with the vehicle controls, and the morphological abnormalities were greater, along with the increase of LA doses.

In accordance with the observations in morphology, MTT assay revealed that cell viability was reduced significantly by 19.4%, 27.8%, 42.2%, 52.5% and 72.1% after treatment with 0.5, 1, 1.5, 2 and 2.5 mm LA for 72 h, respectively, compared with the controls (*P < *0.01; Fig. 2B). The data suggest that LA decreased the viability of lung cancer cells in a dose‐dependent manner.

To further evaluate whether LA induces cell damage, we examined LDH leakage in LA‐treated A549 cells. We observed that medium LDH activity significantly increased by 111.2%, 167.2%, 343.0% and 451.2% after treatment with 0.5, 1, 1.5 and 2 mm LA for 72 h, respectively, compared with the controls (*P < *0.01; Fig. 2C).

**Fig. 3:**
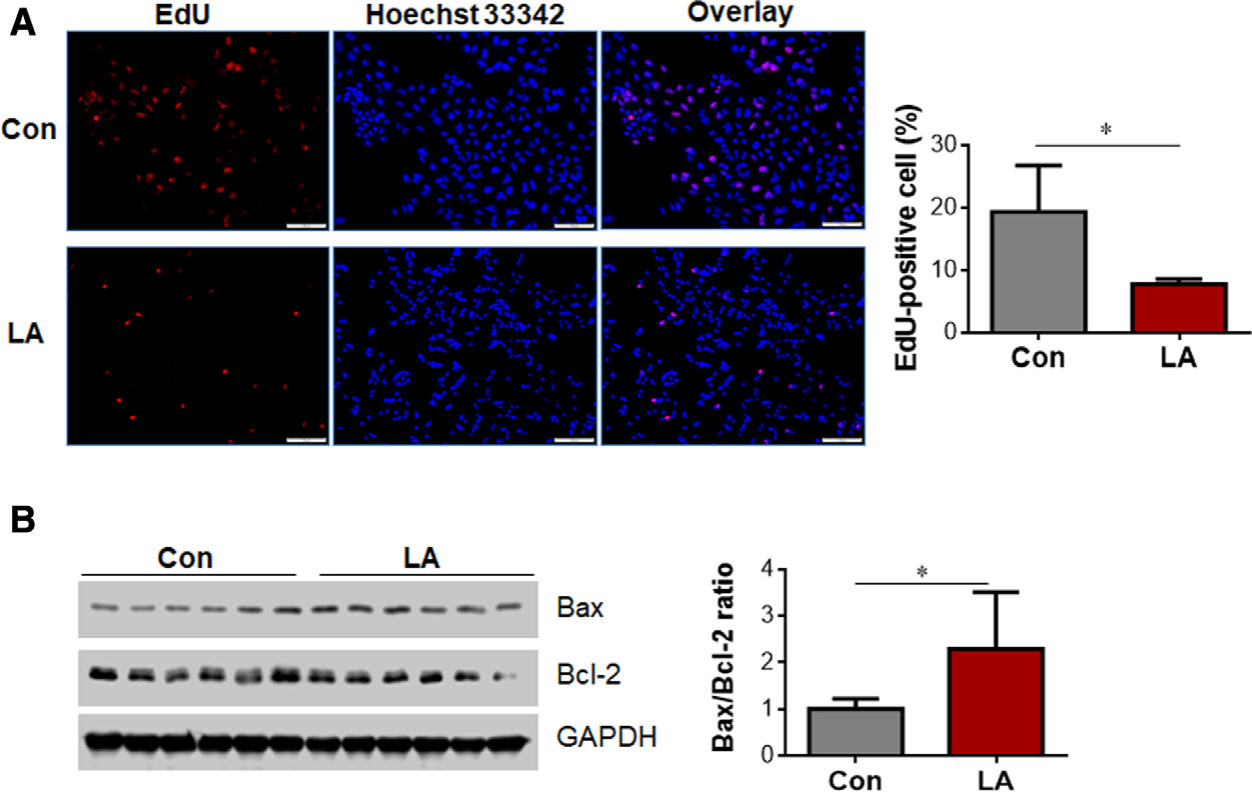
The effect of LA on the proliferation and apoptosis in A549 cells. (A) The effect of LA on the proliferation in A549 cells. A549 cells were treated with LA (0.5 mm) for 72 h. Normal saline‐treated cells served as vehicle controls (Con). Cell proliferation alterations were assessed using EdU labeling, then photographed with a fluorescence microscope at original magnification ×200. Scale bars represent 100 μm. *P < 0.05 by Student’s *t*‐test (versus vehicle controls); error bars represent SD; *n* = 4 per group. (B) Expression of Bcl‐2 and Bax. A549 cells were treated with LA (0.5 mm) for 24 h. Cells were harvested for examination of the Bax/Bcl‐2 ratio. *P < 0.05 by Student’s *t*‐test; error bars represent SD; *n* = 6 per group. GAPDH, glyceraldehyde‐3 phosphate dehydrogenase.

We assessed cell proliferation using EdU labeling. As shown in Fig. 3A, the growth rate of cells significantly decreased by 59.8% after treatment with LA (0.5 mm) for 72 h compared with that in the control group (*P < *0.05).

We also analyzed the effects of LA on the expression of prosurvival protein Bcl‐2 and proapoptotic protein Bax in A549 cells. As shown in Fig. 3B, the Bax/Bcl‐2 ratio increased significantly by 128.7% after treatment with LA (0.5 mm) for 24 h compared with that in control cells (*P < *0.05).

### Human lung cancer tissues show activated autophagy

**Fig. 4:**
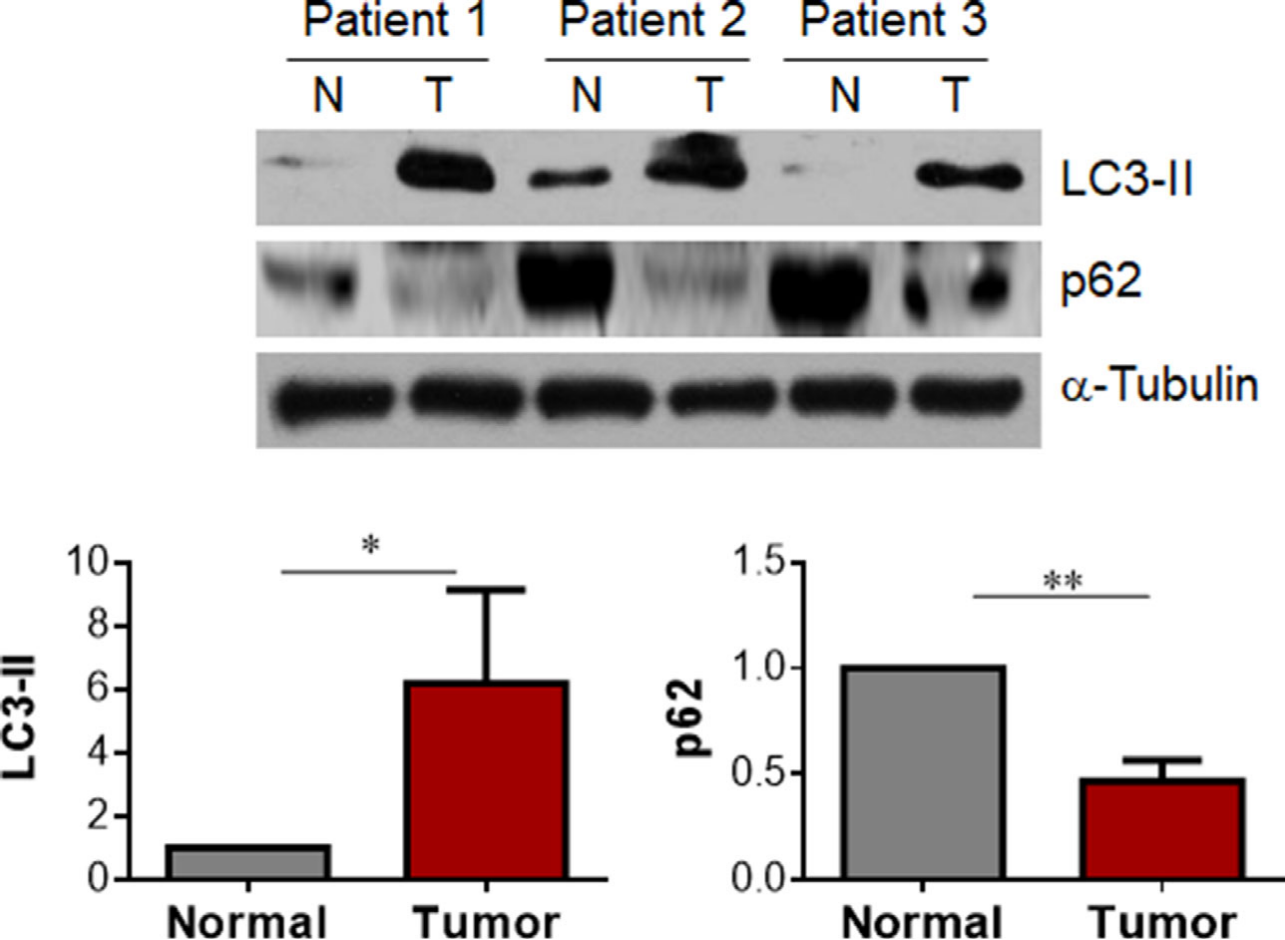
Human lung adenocarcinoma tissues showed activated autophagy. Human lung adenocarcinoma specimen and the matched normal lung tissues were collected from surgical patients. Protein extracts were prepared for immunoblotting analysis against LC3‐II and p62. The blots of α‐Tubulin served as loading controls. **P < 0.01, *P < 0.05 by Student’s *t*‐test; error bars represent SD; *n* = 3 per group. N, normal tissue; T, tumor tissue.

Compelling evidence demonstrates that the activated autophagy facilitates cancer cells to survive from microenvironmental stress and to increase growth [6]. To investigate whether autophagy is involved in the anticancer effect of LA on lung cancer growth, we examined autophagy in human lung adenocarcinoma tissues. Immunoblotting analysis revealed a 517.8% higher level of LC3‐II, a marker for autophagosome formation according to the guidelines for monitoring autophagy [25], in lung tumor tissues compared with the matched normal lung tissues (*P < *0.05; Fig. 4). By striking contrast, human lung cancer tissues showed a significantly decreased level of p62 protein, a protein selectively degraded by autophagy [8, 23, 26, 27] (*P < *0.01; Fig. 4). Taken together, the data indicate an activated autophagy in human lung adenocarcinoma tissues.

### LA inhibits autophagy in A549 lung cancer cells

**Fig. 5:**
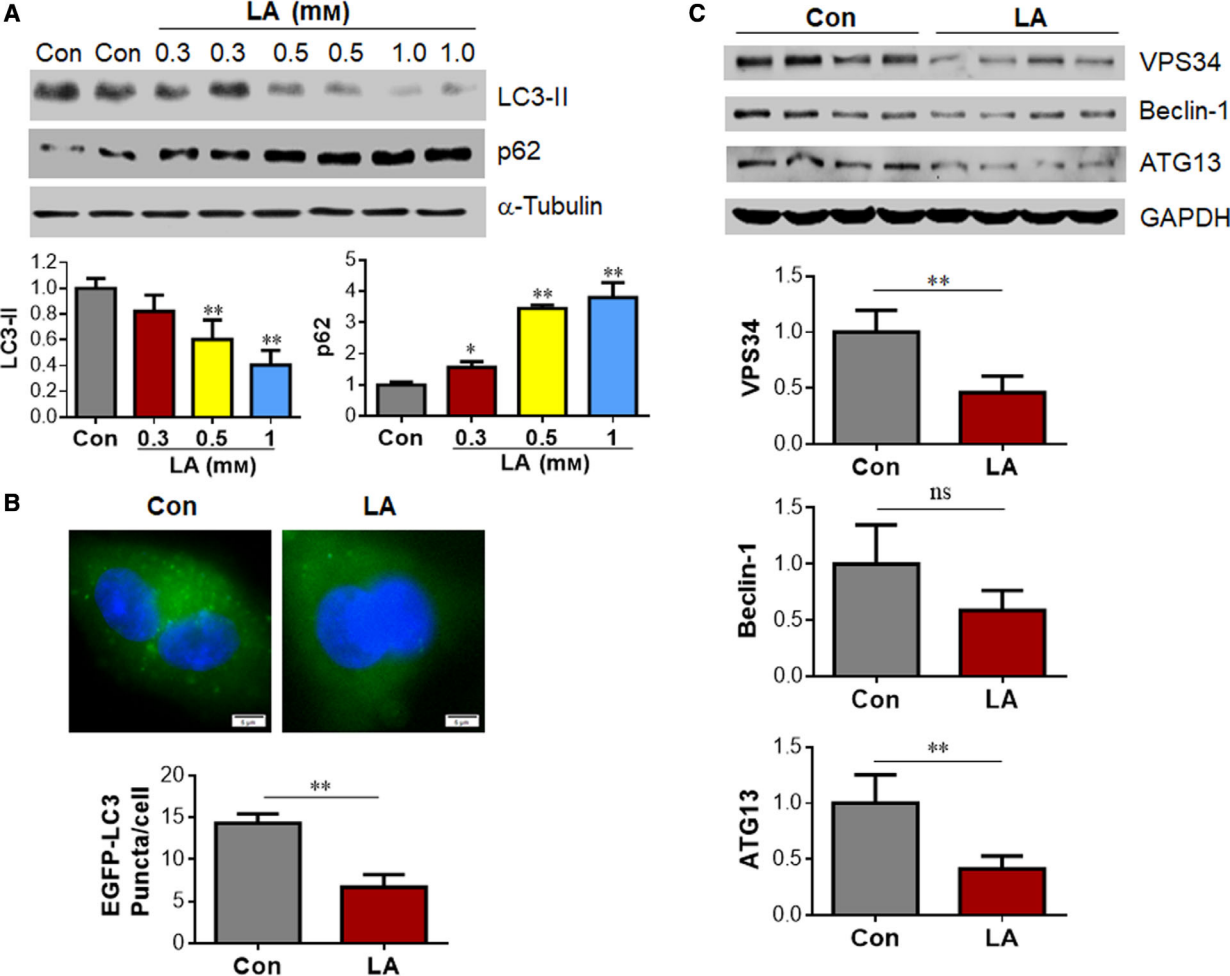
LA inhibited autophagy in A549 lung cancer cells. (A) Protein levels of LC3‐II and p62. After treatment with LA for 24 h at the indicated doses, A549 cells were collected for analyzing LC3‐II and p62 protein abundance. The blots of α‐Tubulin served as loading controls. **P < 0.01, *P < 0.05 by one‐way ANOVA (versus vehicle‐treated controls); error bars represent SD; *n* = 3 for LC3‐II per group and *n* = 4 for p62 per group. (B) EGFP–LC3 punctuation. A549 cells that transfected with the pEGFP–LC3 plasmids were exposed to LA (0.5 mm) for 24 h. Normal saline‐treated cells served as vehicle controls (Con). LC3 punctuation was observed under a fluorescence microscope. Representative images from four independent experiments are shown. Scale bars represent 5 µm. **P < 0.01 by Student’s *t*‐test; error bars represent SD; *n* = 4 per group. (C) Protein levels of VPS34, Beclin‐1 and ATG13. After treatment with LA for 24 h at the indicated doses, A549 cells were collected for analyzing VPS34, Beclin‐1and ATG13 protein abundance. The blots of glyceraldehyde‐3 phosphate dehydrogenase served as loading controls. **P < 0.01 by Student’s *t*‐tests; error bars represent SD; *n* = 4 per group.

To determine whether autophagy is involved in LA‐induced decrease of lung cancer cell survival, we next performed western blot analysis to examine the changes in LC3‐II generation. Although 0.3 mm LA did not significantly change LC3‐II levels in the cells, 0.5 and 1 mm LA significantly reduced LC3‐II protein levels by 39.8% and 59.5%, respectively, compared with the vehicle‐treated controls (*P < *0.01; Fig. 5A). The same results were observed in LC‐II/LC‐I ratios (Fig. S1). To further examine the effect of LA on autophagosome abundance in A549 cells, we examined EGFP–LC3 punctation, a well‐known marker of autophagosomes [8, 23]. To this aim, we transfected A549 cells with plasmid vectors containing pEGFP–LC3. After LA treatment for 24 h, the number of EGFP–LC3 puncta was significantly decreased by 53.3% compared with that in vehicle controls (*P < *0.01; Fig. 5B).

Vps34, Beclin‐1 and ATG13 are the key proteins involved in the initiation of autophagy activation [25]. Immunoblotting showed that 0.5 mm LA significantly reduced Vps34 and ATG13 protein levels by 54.2% and 63.4%, respectively, compared with the vehicle‐treated controls (*P < *0.01; Fig. 5C).

The reduced autophagosome abundance could be attributable to either reduced autophagosome formation or increased autophagosome clearance [8]. We then asked whether the LA‐induced decrease of autophagosome abundance was due to reduced autophagosome formation by examining p62 protein levels, because p62 is selectively incorporated into autophagosomes through directly binding to LC3 and is efficiently degraded by autophagy [8, 23, 26, 27]. Figure 5A shows that 0.5 and 1.0 mm LA significantly increased p62 protein levels by 245.2% and 280.2% in A549 cells, respectively, when compared with the vehicle‐treated controls (*P < *0.01). Altogether, the data suggest that LA inhibits autophagic activity by suppressing autophagosome formation.

### LA activates mTOR signaling

**Fig. 6:**
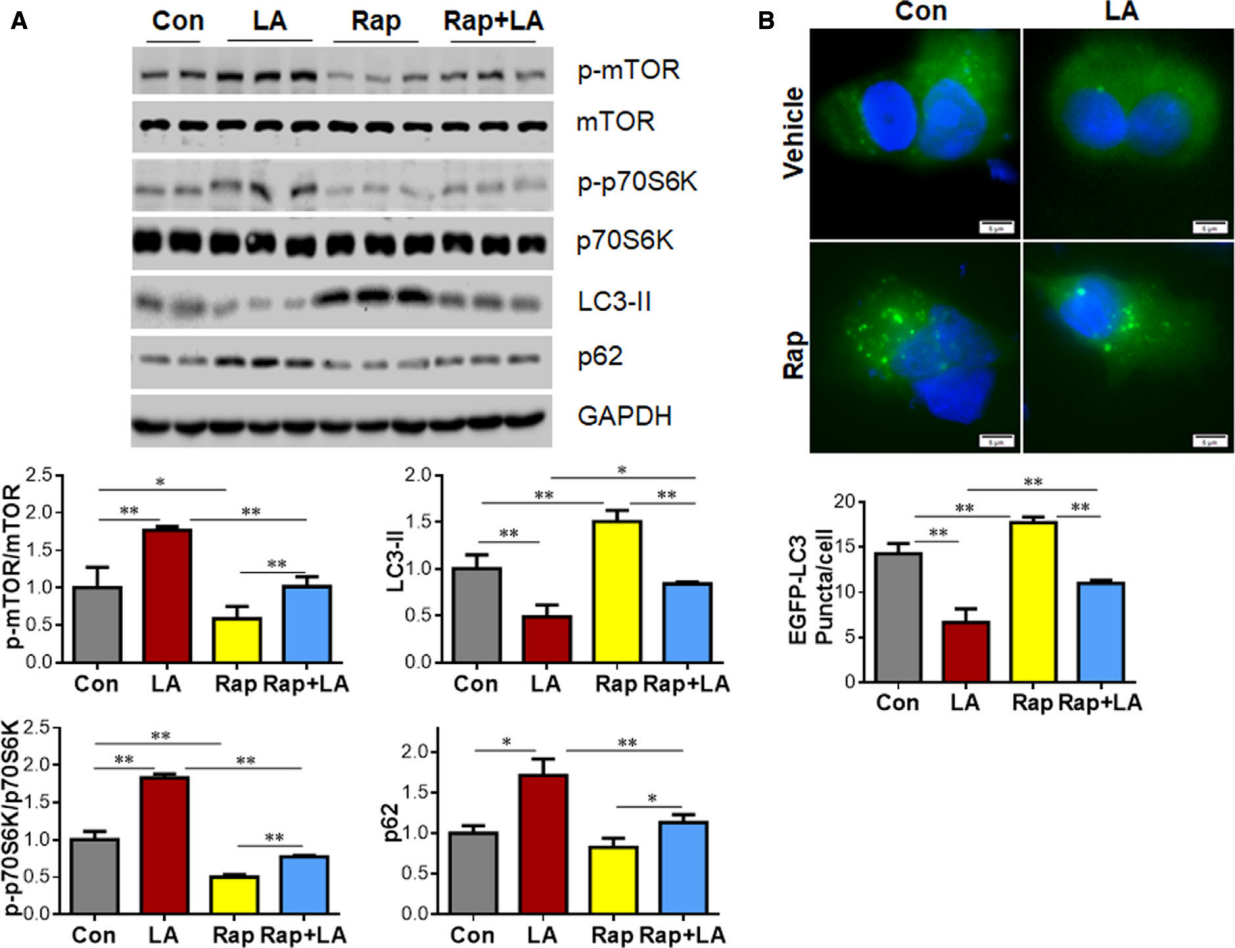
Rap reversed the LA‐induced mTOR activation and autophagy inhibition in A549 cells. (A) Immunoblotting. A549 cells were treated with LA (0.5 mm) for 24 h. Normal saline‐treated cells served as vehicle controls (Con). A549 cells were treated with Rap (500 nm) 1 h before LA (0.5 mm) treatment. Twenty‐four hours after LA treatment, cells were harvested for immunoblotting with the indicated antibodies. **P < 0.01, *P < 0.05 by two‐way ANOVA; error bars represent SD; *n* = 3 per group. (B) EGFP–LC3 punctuation. A549 cells that transfected with the pEGFP–LC3 plasmids were exposed to LA (0.5 mm) for 24 h in the presence or absence of Rap (500 nm). LC3 punctuation was observed under a fluorescence microscope. Representative images from four independent experiments are shown. Scale bars represent 5 µm. **P < 0.01 by two‐way ANOVA; error bars represent SD; *n* = 4 per group.

We next asked how LA reduces autophagosome formation by examining the activation of mTOR/p70S6K signaling, because this signaling pathway plays an important role in the negative regulation of autophagy initiation [8, 27, 28]. Figure 6A shows that LA treatment significantly increased mTOR phosphorylation level by 76.9% compared with that in vehicle controls (*P < *0.01). Also, the phosphorylation level of p70S6K, a downstream target of mTOR, was increased by 83.2% when compared with controls (*P < *0.01). Akt, an upstream kinase for mTOR phosphorylation, showed the same changes by LA treatment (Fig. S2). The data suggest that the LA‐induced inhibition of autophagy might involve the activation of mTOR‐dependent signaling.

### Pharmacological inhibition of mTOR reversed the LA‐induced inhibition of autophagy in A549 lung cancer cells

Considering that mTOR could inhibit autophagy activation [8, 27, 28], we then asked whether the LA‐induced inhibition of autophagy and reduction of cell viability are mediated by mTOR activation. To this end, we treated A549 cells with Rap, a widely used specific inhibitor of mTOR, 1 h before LA administration. As expected, Rap administration decreased phosphorylation levels of mTOR and its downstream target p70S6K in A549 cells compared with the vehicle‐treated controls (Rap versus control, *P < *0.01 or *P < *0.05; Fig. 6A). Moreover, the LA‐induced increases of mTOR and p70S6K phosphorylation levels were reversed by Rap pretreatment (Rap+LA versus LA, *P < *0.01; Fig. 6A). Importantly, the Rap pretreatment prevented the LA‐induced decrease of LC3‐II level in A549 cells (Rap+LA versus LA, *P < *0.05; Fig. 6A). By contrast, the LA‐induced increase of p62 protein abundance was prevented by Rap pretreatment (Rap+LA versus LA, *P < *0.01; Fig. 6A). To further determine the role of mTOR in LA‐induced autophagosome abundance, we examined EGFP–LC3 punctation in LA‐treated A549 cells. Consistent with the results in LC3‐II contents (Fig. 6A), LA‐treated cells showed a higher number of EGFP–LC3 puncta in the presence of Rap than that in the absence of Rap (Rap+LA versus LA, *P < *0.01; Fig. 6B). Collectively, the data suggest that the LA‐induced autophagy inhibition depended on mTOR activation.

### Pharmacological inhibition of mTOR reversed the LA‐induced suppression of A549 cell viability

**Fig. 7:**
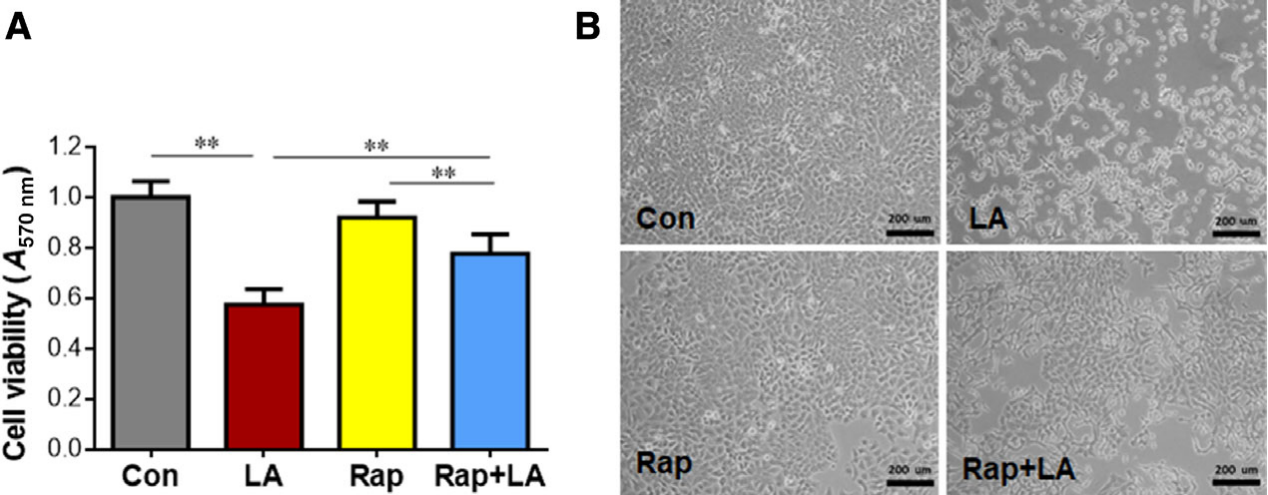
Rap reversed the LA‐induced reduction of cell viability. (A) MTT assay. A549 cells were treated with Rap (500 nm) 1 h before LA (0.5 mm) treatment. Seventy‐two hours after LA treatment, cell viability was analyzed by MTT assay. **P < 0.01 by two‐way ANOVA; error bars represent SD; *n* = 6 per group. (B) Morphological examination. A549 cells were treated with Rap (500 nm) 1 h before LA (0.5 mm) treatment. Seventy‐two hours after LA treatment, cell morphological alterations were observed using a phase‐contrast light microscope. Scale bars represent 200 µm. *n* = 3 per group.

We then asked whether the LA‐induced inhibition of A549 cell viability is mediated by mTOR. Solely administration with Rap did not significantly change the cell viability (Rap versus control; Fig. 7A). However, in the presence of Rap, LA‐treated cells exhibited 35.3% more viability when compared with the LA‐treated cells in the absence of Rap (Rap+LA versus LA, *P < *0.01). Accordingly, morphological examination showed that the LA‐induced cell morphological abnormalities and cell density reduction were obviously diminished by Rap preadministration (Rap+LA versus LA; Fig. 7B).

### Synergistic effect of LA with the autophagy inhibitor CQ on suppression of A549 cell viability

**Fig. 8:**
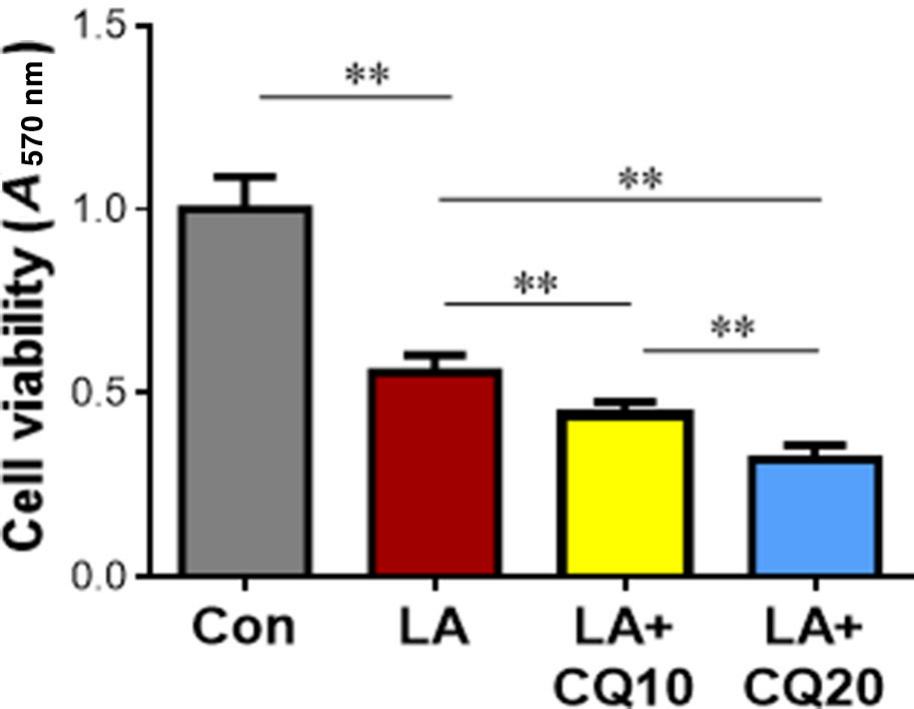
Synergistic effects of LA with CQ on the inhibition of cell viability. A549 cells were treated with autophagic inhibitor CQ (10 or 20 µm) 1 h before LA (0.5 mm) exposure. Normal saline‐treated cells served as vehicle controls (Con). Seventy‐two hours after LA treatment, cell viability was evaluated using MTT assay. **P < 0.01 by one‐way ANOVA; error bars represent SD; *n* = 4 per group.

Finally, we examined whether LA suppresses A549 cell viability synergistically with CQ, a widely used autophagy inhibitor [8]. Notably, CQ enhanced the LA‐induced reduction of cell viability by 20.9% and 42.7% at the doses of 10 and 20 μm, respectively, compared with the cells solely treated with LA (*P < *0.01; Fig. 8). The data suggest a synergistic effect of LA with autophagy inhibitor on the suppression of A549 cell viability.

## Discussion

The major finding of this study is that LA, a compound found in the human diet that has been used for treating diabetic complications in humans, limited lung cancer growth in mice. The anti‐lung cancer effect of LA was mediated through the mTOR‐mediated inhibition of autophagy. Our data suggest that besides the treatment for diabetic complications, LA might serve as an alternative therapeutic approach for lung cancer of humans.

We observed that oral administration suppressed growth of lung tumor in mice. LA is a compound found in the human diet as a naturally occurring co‐actor for metabolic enzymes. Clinically, LA has been used in treating symptomatic peripheral neuropathy, cardiac autonomic neuropathy, as well as other complications in patients with diabetes for more than 20 years [18, 29]. Moreover, LA is used in the management of other disorders, such as Alzheimer's disease and Parkinson's disease, in clinical and experimental studies. Interestingly, recent *in vitro* studies have demonstrated that LA induces death or inhibits proliferation in cancer cells, including hepatoma cells, colon cancer cells and acute T cell leukemia [17, 30, 31]. However, little is known about whether LA could impact tumor growth in intact animals. To address this question, we orally treated nude mice at the day of receiving human lung cancer A549 cell implantation. We observed that after oral administration for 18 days, LA significantly decreased tumor nodule numbers and tumor burdens in lungs of mice, respectively, when compared with that in normal saline‐treated control mice. Our *in vivo* data clearly indicate that LA suppressed lung cancer progression in intact animals.

To dissect the underlying mechanism for how LA suppresses lung tumor growth in mice, we performed *in vitro* analysis in human lung cancer A549 cell cultures. We found that the cell viability was significantly reduced by LA exposure in a dose‐dependent manner. Moreover, the cell proliferation, as indicated by EdU incorporation, was inhibited by LA treatment. However, the expressions of cycling genes, such as *c‐Myc* and *Cyclin D1*, were upregulated by LA (Fig. S3), suggesting that the LA‐induced inhibition of proliferation is regulated by another mechanism, rather than by involvement of c‐Myc and Cyclin D1. The Bax/Bcl‐2 ratio increased in LA‐treated A549 cells, suggesting that the cells might undergo death after LA treatment. In support of this, the LA‐treated A549 cells showed a round, shrunken shape, lost integrity and detachment from culture dishes. Collectively, the data indicate that the LA‐induced decrease of viability in A549 cells involved cell injury.

Previous evidence has demonstrated that LA induces apoptosis through increasing mitochondrial $O_{2}^{-\cdot }$ production, Akt inhibition and activating p27Kip‐dependent cell‐cycle arrest in human colon cancer cells, hepatoma cells and squamous cell carcinoma cells [17, 30, 31]. By contrast with these observations, we found an upregulation of cell‐cycle‐related proteins (c‐Myc and Cyclin D1) in LA‐treated A549 cells in this study (Fig. S3) and found an activation of Akt by LA in this study (Fig. S2) and in previous reports [19], suggesting that the LA‐induced decrease of A549 cell viability may be through the mechanisms different from the aforementioned studies in human colon cancer cells, hepatoma cells and squamous cell carcinoma cells [17, 30, 31]. Intriguingly, we found higher autophagic activity in human lung cancers as reflected by increased LC3‐II abundance together with decreased p62 protein content. In contrast, a reduced autophagy was detected in LA‐treated A549 cells as reflected by decreased LC3‐II generation, reduced LC3 punctuation, decreased VPS34 and ATG13 expression, and increased p62 protein levels. Autophagy is an evolutionarily conserved catabolic process and serves as the major intracellular degradation system. In normal cells, autophagy has been shown to suppress malignant transformation. However, once malignant transformation has occurred, autophagy can promote tumor progression and resistance to therapy [4]. Therefore, autophagy is considered a promising therapeutic candidate for cancer treatment. Taken together, our data indicate that the autophagy inhibition may be involved in the LA‐induced anti‐lung cancer effects.

Autophagosome formation is the first step of the autophagic process. Evidence has demonstrated that mTOR plays a central role in the negative regulation of autophagosome formation by integrating various signaling molecule networks [11]. Interestingly, we found an activation of mTOR and its downstream target p70S6K in A549 lung cancer cells after LA exposure, suggesting that LA may suppress the autophagosome formation in a mTOR‐dependent manner. Indeed, the LA‐treated cells showed decreased LC3 punctuation and increased p62 protein levels. When taking into account that p62 is a protein that is selectively cleared by the autophagy‐degraded pathway [8], our data show that LA inhibited autophagy at an early phase. To determine the role of mTOR‐mediated autophagy inhibition in the LA‐induced suppression of lung cancer cell viability, we used Rap, a widely used mTOR‐selective inhibitor, in the experiments. After inhibition of mTOR with Rap, the LA‐induced autophagy inhibition and cell viability reduction were reversed. Moreover, we found a synergistic effect of LA with the autophagy inhibitor CQ on the inhibition of A549 cell viability. Altogether, our data suggest that LA suppressed lung cancer cell viability through inhibition of autophagy in an mTOR‐dependent manner.

In summary, we provide clear evidence from both *in vivo* and *in vitro* experiments that LA suppressed lung cancer growth and lung cancer cell viability. This anti‐lung cancer effect of LA was mediated by the mTOR‐mediated autophagy inhibition. When taken into account that LA has been used in the treatment of human diabetic complications for many years and shown no obvious adverse effects, LA might represent a meaningful therapeutic approach for lung cancer in humans.

## Conflict of interest

The authors declare no conflict of interest.

## Author contributions

ZD and XC conceived the project and designed the study. PP, XZ, TQ, HC and QK performed the human, animal and cell experiments. LL analyzed data. ZD and PP wrote the manuscript. All authors read and approved the final manuscript.

## Supporting information


**Fig. S1.** LA decreased the LC3‐II/LC3‐I ratio. After treatment with LA (0.5 mM) for 24 h, A549 cells were collected for analyzing the expression of LC3‐I and LC3‐II. **P < 0.01 by Student’s *t*‐test; error bars represent SD; *n* = 4 per group.

**Fig. S2.** LA increased the Akt phosphorylation level. A549 cells were treated with LA (0.5 mM) for 24 h. Normal saline‐treated cells served as vehicle controls (Con). Cells were harvested for immunoblotting with the indicated antibodies. **P < 0.01 by Student’s *t*‐test; error bars represent SD; *n* = 4 per group.

**Fig. S3.** LA increased Cyclin D1 and C‐Myc expression. After treatment with LA (0.5 mM) for 24 h, A549 cells were collected for analyzing the expression of Cyclin D1 and c‐Myc. **P < 0.01, *P < 0.05 by Student’s *t*‐test; error bars represent SD; *n* = 6 per group.
