# Supplementary figures and images for: Alpha‐lipoic acid inhibits lung cancer growth via mTOR‐mediated autophagy inhibition
Source: FEBS Open Bio. 2020 Mar 18;10(4):607–18. doi: 10.1002/2211-5463.12820 (PMC7137803; doi:10.1002/2211-5463.12820)

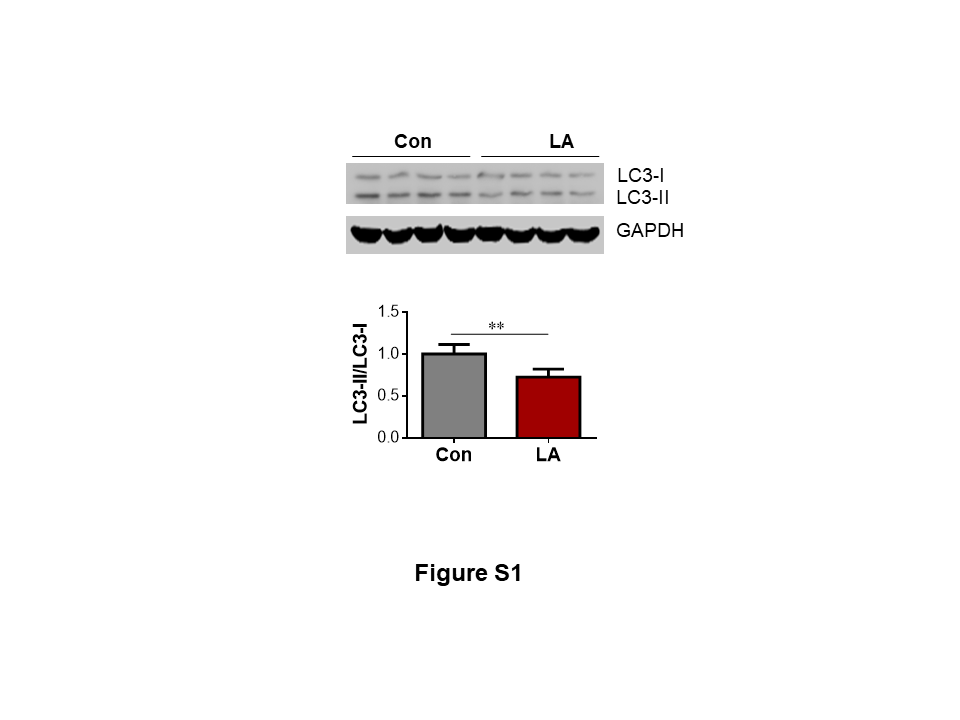

Supplement: Supplementary file 1 — Fig. S1. LA decreased the LC3‐II/LC3‐I ratio. After treatment with LA (0.5 mM) for 24 h, A549 cells were collected for analyzing the expression of LC3‐I and LC3‐II. **P < 0.01 by Student’s t‐test; error bars represent SD; n = 4 per group. [file FEB4-10-607-s001.TIF]

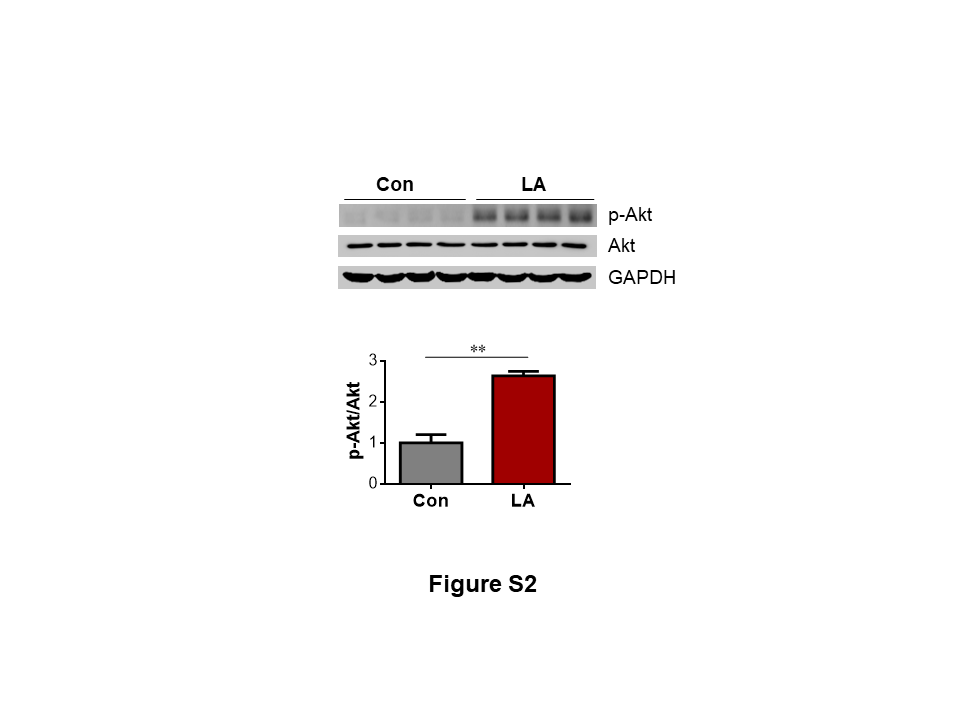

Supplement: Supplementary file 2 — Fig. S2. LA increased the Akt phosphorylation level. A549 cells were treated with LA (0.5 mM) for 24 h. Normal saline‐treated cells served as vehicle controls (Con). Cells were harvested for immunoblotting with the indicated antibodies. **P < 0.01 by Student’s t‐test; error bars represent SD; n = 4 per group. [file FEB4-10-607-s002.TIF]

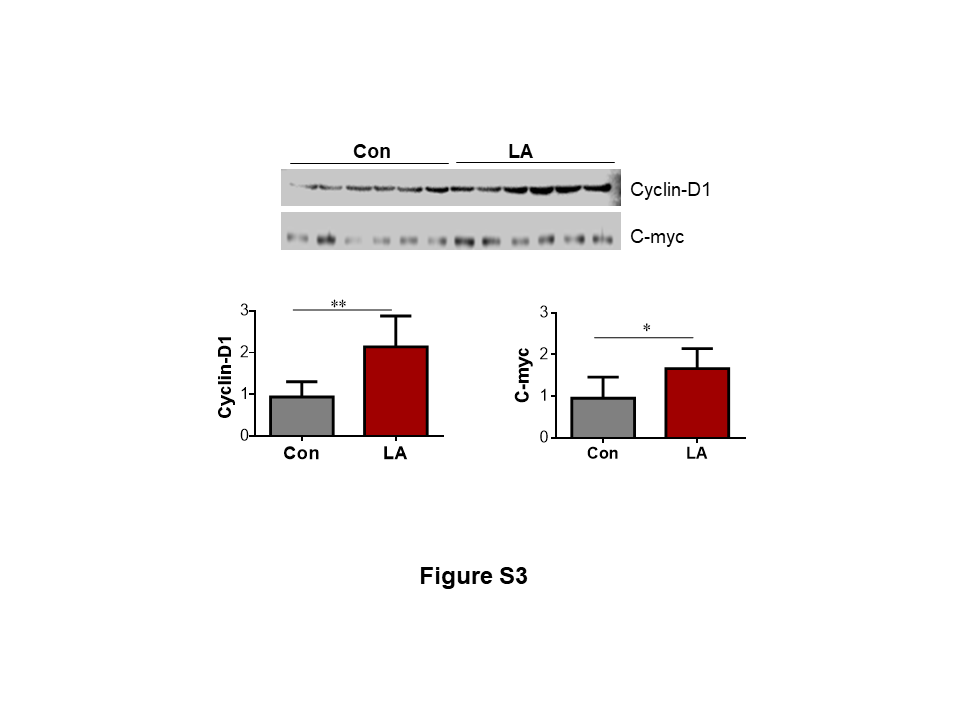

Supplement: Supplementary file 3 — Fig. S3. LA increased Cyclin D1 and C‐Myc expression. After treatment with LA (0.5 mM) for 24 h, A549 cells were collected for analyzing the expression of Cyclin D1 and c‐Myc. **P < 0.01, *P < 0.05 by Student’s t‐test; error bars represent SD; n = 6 per group. [file FEB4-10-607-s003.TIF]
