# Supplementary material for: Alpha‐lipoic acid inhibits lung cancer growth via mTOR‐mediated autophagy inhibition
Source: FEBS Open Bio. 2020 Mar 18;10(4):607–18. doi: 10.1002/2211-5463.12820 (PMC7137803; doi:10.1002/2211-5463.12820)
Supplement: Supplementary file 4 [file FEB4-10-607-s004.docx]

**Supplemental Figure Legends**

**Figure S1. LA decreased the ratio of LC3-II/LC3-I.**

Following treatment with LA (0.5 mM) for 24 h, A549 cells were collected for analyzing the expression of LC3-I and LC3-II. ** *P <* 0.01 by student’s *t*-test, Error bars represent SD, n = 4 per group.

**Figure S2. LA increased Akt phosphorylation level**

A549 cells were treated with LA (0.5 mM) for 24 h. Normal saline-treated cells served as vehicle controls (Con). Cells were harvested for immunoblotting with the indicated antibodies. ***P <* 0.01 by student’s *t*-test, Error bars represent SD, n = 4 per group.

**Figure S3. LA increased** **Cyclin-D1 and C-Myc expression.**

Following treatment with LA (0.5 mM) for 24 h, A549 cells were collected for analyzing the expression of Cyclin-D1 and C-Myc. ** *P <* 0.01 and * *P <* 0.05 by student’s *t*-test, Error bars represent SD, n = 6 per group.
